# O Prognóstico da Doença Arterial Coronariana em um Hospital Público no Brasil: Achado do Estudo ERICO

**DOI:** 10.36660/abc.20200399

**Published:** 2021-09-16

**Authors:** Tatiana Cristina Bruno, Marcio Sommer Bittencourt, Alessandra V. L. Quidim, Itamar Santos, Paulo Lotufo, Isabela Bensenor, Alessandra Goulart

**Affiliations:** 1 Universidade de São Paulo Hospital Universitário de São Paulo Centro de Pesquisa Clínica e Epidemiológica São Paulo SP Brasil Universidade de São Paulo, Hospital Universitário de São Paulo - Centro de Pesquisa Clínica e Epidemiológica, São Paulo, SP - Brasil

**Keywords:** Sobrevivência, Mortalidade, Síndrome Coronariana Aguda, Doença Arterial Coronariana, Hospital Público, Epidemiologia

## Abstract

**Fundamento:**

O prognóstico de longo prazo pós síndrome coronária aguda (SCA) no cuidado secundário não é bem conhecido. A gravidade da doença arterial coronariana (DAC) como preditor de mortalidade no longo prazo foi avaliada em um hospital público no Brasil.

**Objetivo:**

O objetivo deste estudo foi comparar o prognóstico de curto e longo prazo após um evento de SCA, de acordo com a gravidade da doença obstrutiva, em pacientes atendidos em um hospital público secundário para um coorte prospectivo sobre DAC no Brasil (o Estudo de Registro de Insuficiência Coronariana, estudo ERICO)

**Métodos:**

Foram realizadas análises de sobrevida por curvas de Kaplan-Meier e modelo de risco proporcional de Cox [razão de risco (RR) com o respectivo intervalo de confiança (IC) de 95% para avaliar mortalidade cumulativa global, por DCV e DAC, de acordo com a obstrução arterial coronária: sem obstrução (grupo de referência), doença de um vaso, doença de dois vasos, doença de múltiplos vasos] entre 800 adultos do estudo ERICO durante 4 anos de monitoramento. As RR são apresentadas como dados brutos e posteriormente padronizadas quanto a possíveis fatores de confusão, no período de 180 dias até 4 anos de monitoramento após a SCA. O p-valor <0.05 foi considerado estatisticamente significativo.

**Resultados:**

Taxas de sobrevida mais baixas foram detectadas entre indivíduos com a doença de múltiplos vasos (global, DCV e DAC, p de teste de Log-rank <0,0001). Depois da padronização multivariada, a doença de múltiplos vasos [RR; 2,33 (IC 95%; 1,10-4,95)] e doença de um vaso obstruído [RR; 2,44 (IC 95%; 1,11-5,34)] tiveram o risco mais alto de mortalidade global comparadas aos índices dos sujeitos sem obstrução no monitoramento de 4 anos.

**Conclusões:**

Não só os pacientes com doença de múltiplos vasos como também os com doença de um vaso tiveram alto risco de mortalidade no longo prazo pós-SCA. Esses achados destacam a importância de se ter uma abordagem melhor no tratamento e no controle de fatores de risco cardiovascular, mesmo em indivíduos com risco aparentemente baixo, atendidos em cuidado secundário.

## Introdução

Doenças cardiovasculares (DCV), especialmente a doença arterial coronariana (DAC), ainda são a principal causa de mortalidade em todo o mundo, inclusive no Brasil.^1 , 2^ A síndrome coronária aguda (SCA), que inclui angina instável (AI), infarto agudo do miocárdio (IAM) com supradesnivelamento do segmento ST (STEMI) e sem supradesnivelamento do segmento ST (NSTEMI), representa uma carga significativa em países com renda baixa e média, incluindo o Brasil.^3^ Estatísticas nacionais revelam uma carga mais alta de mortalidade entre os que estão nas camadas sociais mais baixas, população trabalhadora e mais jovem, em comparação a populações mais ricas.^2 , 3^ A maior parte dos dados que relatou um prognóstico de longo prazo em DAC vem de estudos prospectivos realizados em países desenvolvidos.^4 - 6^ Nesses estudos de centros especializados com unidades de cardiologia com nível de cuidado terciário, taxas de mortalidade em longo prazo mais altas foram descritas entre aqueles com número mais alto de artérias obstruídas e gravidade de DAC comparados com os pacientes sem obstrução (<50%).^7 - 9^ Nesse cenário, a sobrevida em longo prazo após um evento SCA ainda não é bem conhecida entre pacientes avaliados no cuidado secundário ou primário. Além disso, a falta de acesso a abordagens cardiológicas mais especializadas e tratamento após um evento coronário agudo é um enorme problema de saúde, especialmente em países em desenvolvimento. Por exemplo, estudos anteriores já indicaram um prognóstico pior em pacientes com DAC admitidos em cuidado primário e secundário, sem indicação para cuidado especializado.^10 - 12^ O mesmo se aplica ao Brasil, onde as dificuldades de acessar o cuidado terciário também parecem ser responsáveis por índices de mortalidade mais altos.^13^ Portanto, o objetivo deste estudo foi comparar o prognóstico de curto e longo prazo após um evento de SCA de acordo com a gravidade da doença obstrutiva em pacientes atendidos em um hospital público secundário de um coorte prospectivo sobre DAC no Brasil (o Estudo de Registro de Insuficiência Coronariana, estudo ERICO)

## Métodos

### Desenho e população da amostra

Todos os pacientes eram participantes do estudo ERICO, um coorte prospectivo de indivíduos com SCA recrutados no Hospital Universitário da Universidade de São Paulo (HU-USP) de fevereiro de 2009 a dezembro de 2013. Mais detalhes sobre o estudo ERICO são apresentados em outro lugar.^14^

Resumidamente, o estudo ERICO é um coorte contínuo do HU-USP, um hospital público de cuidado secundário com 260 leitos, localizado no bairro do Butantã, que tinha uma população de 428.000 habitantes em 2010.^15 , 16^ Embora o Butantã tenha alguns indicadores socioeconômicos acima da média da cidade (por exemplo, renda familiar média), é uma região caracterizada por grande desigualdade.^16^

Aqui, avaliamos todos os participantes (n=800/1085, 73,7%) admitidos na emergência do HU-USP, como SCA confirmada, submetidos a angiografia invasiva para o diagnóstico de obstrução coronária, e decisão clínica posterior à fase aguda (tratamento exclusivamente clínico, intervenção coronária percutânea (ICP) ou enxerto de bypass na artéria coronária (CABG)). Todos os exames foram realizados em nossa principal clínica de referência durante a fase aguda do evento coronariano, o Instituto do Coração (InCor), um centro de referência em cardiologia a aproximadamente oito quilômetros do HU-USP. Como o HU-USP não é um hospital especializado, não há disponibilidade de procedimentos de cateterismo cardíaco ou de CABG.

### Definição de Síndrome Coronária Aguda (SCA)

Todos os pacientes com suspeita de SCA na emergência do HU-USP passaram por triagem para participar do estudo ERICO. Os critérios de qualificação para participar do ERICO exigem que o paciente seja diagnosticado como tendo tido infarto do miocárdio com supradesnivelamento do segmento ST (STEMI), sem supradesnivelamento do segmento ST (NSTEMI) ou angina instável (AI). Os critérios usados para definir a SCA foram:^14 - 17^

1. Infarto do miocárdio: a presença de sintomas consistentes com isquemia cardíaca em um período de 24 horas antes da entrada no hospital e níveis de troponina I acima do 99º percentil com um coeficiente de variação específico de teste <10%.
  1a) Infarto do miocárdio com supradesnivelamento do segmento ST> presença de critérios para doença arterial coronariana (DAC) mais um dos seguintes: supradesnivelamento do segmento ST persistente maior ou igual a 1 mm em duas derivações eletrocardiográficas contíguas ou a presença de um novo, ou presumidamente novo, bloqueio de ramo esquerdo.
  1b) Infarto do miocárdio sem supradesnivelamento do segmento ST: presença de critérios para infarto do miocárdio, mas não infarto do miocárdio com supradesnivelamento do segmento ST
2. Angina instável: sintomas consistentes com isquemia cardíaca 24 horas antes da admissão hospitalar, ausência de critérios de infarto do miocárdio, e pelo menos um dos seguintes: histórico de doença arterial coronariana; teste de estratificação de doença coronária positivo (invasivo ou não invasivo); alterações temporárias no segmento ST maiores ou iguais a 0,5 mm em duas derivações contíguas, nova inversão de onda T maior ou igual a 1 mm e/ou pseudonormalização de ondas T invertidas anteriormente; troponina I maior ou igual a 0,4 ng/ml (o que garante que os níveis de troponina I estão acima do 99º percentil independentemente do kit utilizado); ou concordância diagnóstica de dois médicos independentes.

### Classificação da doença arterial coronariana

A classificação da doença coronária foi baseada na presença de obstrução ≥50% de pelo menos 1 artéria coronária principal ou qualquer uma de suas derivações: artéria descendente anterior (DA), artéria circunflexa (CX) e artéria coronária direita (ACD). As seguintes categorias de obstrução coronária consistiram em: Grupo 1: sem obstrução, quando todos os vasos tinham <50% de obstrução; Grupo 2: doença de um vaso, quando ≥50% de obstrução estava presente em uma artéria coronária principal ou qualquer um de seus ramos; Grupo 3: doença de dois vasos, quando havia ≥50% de obstrução em duas artérias coronárias principais ou em seus ramos principais; e Grupo 4: doença de múltiplos vasos, com obstrução de todas as três coronárias principais (ou seus principais ramos), ou obstrução ≥50% da artéria coronária esquerda principal (ACE), ou presença de enxerto de bypass na artéria coronária (CABG).

### Protocolo do estudo

Na admissão hospitalar por SCA, após a assinatura do consentimento informado, todos os participantes forneceram informações de linha de base com base em questionários padronizados que incluíram dados sociodemográficos, principais fatores de risco cardiovascular (hipertensão, diabetes, obesidade, dislipidemia, tabagismo, histórico familiar ou pessoal de doença arterial coronariana, sedentarismo, uso de cocaína ou menopausa) e o uso anterior de medicamentos. As condições clínicas foram autorrelatadas.

Três médicos foram independentemente responsáveis por analisar as informações e validar os casos de SCA. O protocolo do estudo também incluiu amostra sanguínea para exames laboratoriais, tais como: troponina I, creatina quinase MB, hemograma e perfil lipídico (incluindo colesterol total, HDL e LDL- colesterol (C), e triglicérides).

Depois de 30 dias do evento agudo, todos os participantes foram convidados a atualizar suas informações sobre riscos cardiovasculares. Aos seis meses e anualmente após o evento inicial, os pacientes foram contactados por telefone para atualizar suas informações, seu status vital, seu histórico cardiovascular e o uso de medicamentos. Sempre que um paciente relatava um novo possível evento de SCA, era iniciada uma investigação para coletar mais informações. O ERICO foi descrito em outro lugar.^14^

## Resultados

Informações sobre três desfechos fatais, mortalidade global, por DCV ou por DAC, foram registradas pelo estudo ERICO. O status vital foi atualizado por meio de prontuários médicos e certidões de óbito. Os dados de mortalidade foram confirmados por certidões oficiais de óbito em colaboração com o sistema de estatísticas de saúde da cidade de São Paulo (PRO-AIM, Programa de Aprimoramento das Informações de Mortalidade do município de São Paulo), com os órgãos estaduais de saúde (SEADE - Fundação Sistema Estadual de Análise de Dados Estatísticos), e com o Ministério da Saúde do Brasil. A equipe de pesquisa preparou, regularmente, uma lista de indivíduos que foram relatados como mortos ou com quem o contato foi perdido. Os órgãos de saúde estaduais e municipais pesquisaram em seus bancos de dados certidões de óbito relatando resultados à equipe de pesquisa do estudo ERICO. No presente estudo, a causa básica da morte foi utilizada. Dois médicos analisaram independentemente as certidões de nascimento e, quando necessário, a causa da morte subjacente foi reclassificada. Caso houvesse discordância entre eles, um terceiro médico realizava a análise da certidão de óbito, seguida de discussão e decisão por consenso. A causa de morte dos participantes era definida como causa cardiovascular (“mortalidade cardiovascular”) quando poderia ter sido definida como “Doença do sistema circulatório” de acordo com a Classificação Internacional de Doenças (CID-10), capítulo IX, ou se a causa morte fosse identificada de acordo com o código R57.0 do CID-10, “Choque cardiogênico”. Cada evento identificado foi adjudicado utilizando-se critérios internacionais pré-definidos.^18 , 19^ A mortalidade dos participantes foi classificada como “mortalidade pós-IM”, sempre que uma DAC fatal foi identificada como a principal causa de morte. Para a causa de morte por DAC, usou-se a definição de infarto do miocárdio (I21.X), também presente no Capítulo IX sobre doenças circulatórias, CID-10. A mortalidade global se refere às mortes independentemente das causas subjacentes.

O protocolo do estudo foi aprovado pelo Comitê de Análise Institucional relacionado a pesquisa em seres humanos. Todos os sujeitos forneceram um formulário de consentimento informado por escrito para o estudo.

### Análise estatística

As análises descritivas dos participantes do ERICO foram apresentadas de acordo com os grupos pré-definidos de obstrução coronária descritos acima. As variáveis categóricas, apresentadas como frequências absolutas e relativas, foram analisadas pelo teste qui-quadrado. Como não se observou distribuição paramétrica pelo teste de normalidade de Kolmogorov-Smirnov, as variáveis contínuas são apresentadas como valores medianos com a respectiva faixa interquartil (FIQ), e a distribuição entre os subgrupos de obstrução coronária foi comparada utilizando-se os testes de Kruskal-Wallis.

Foram realizadas análises de sobrevida por curvas de Kaplan-Meier^20^ e modelo de risco proporcional de Cox (razão de risco (RR) com o respectivo intervalo de confiança (IC de 95%)^21^ para avaliar mortalidade cumulativa global, por DCV e DAC, de acordo com o número de artérias coronárias principais obstruídas, ou qualquer um de seus ramos principais (sem obstrução (grupo de referência), doença de um vaso, doença de dois vasos, doença de múltiplos vasos). Para todos os pacientes na amostra houve um período de monitoramento de 7 anos, com o tempo médio de acompanhamento de 1.460 dias, o correspondente a 4 anos. Portanto, optou-se por fazer a análise de regressão de Cox e a Razão de risco aos 180 dias e anualmente até 4 anos após um evento agudo. Os modelos de regressão de Cox foram calculados da seguinte maneira: brutos, padronizados para idade e sexo, e modelo completo padronizado com histórico de DAC prévia, subtipo de SCA (AI, NSTEMI, STEMI), tabagismo (ex-fumante, fumante e nunca fumou), hipertensão, diabetes, dislipidemia e tipo de procedimento realizado (tratamento médico, percutâneo ou cirúrgico). Modelos adicionais padronizados quanto ao colesterol LDL, uso anterior de aspirina, medicamentos redutores de lipídeos, inibidores de enzima conversora da angiotensina (ECA), e β-bloqueadores também foram avaliados.

Todos os testes foram bicaudais, com uma significância de <0,05. Todas as análises estatísticas foram realizadas utilizando-se o programa SPSS® Statistics versão 25.0 disponibilizado pela IBM®.

## Resultados

### Casuística

Dos 800 participantes que passaram por angiografia invasiva (de fevereiro de 2009 a dezembro de 2013), 343 (42,9%) foram submetidos a um tratamento conservador, incluindo pelo menos três dos seguintes medicamentos: aspirina, β bloqueador, inibidor ECA ou inibidor de enzima conversora da angiotensina II, e medicamentos redutores de lipídeos (estatinas ou fibratos). Entre os que faziam o tratamento conservador, 15 (4,4%) sofreram trombólise química. Em relação às estratégias terapêuticas invasivas, 400 participantes (50,0%) passaram por intervenção coronária percutânea (ICP), com implante de stent (75,8% com stent de metal, 13,3% com angioplastia por balão, 10,9% com stent farmacológico), e 57 (7,2%) passaram por CABG.

### Características clínicas e sociodemográficas

As características clínicas e sociodemográficas de acordo com o número de principais artérias coronárias obstruídas são apresentadas na Tabela 1. Em relação à presença de principais artérias coronárias obstruídas, foram: 107 (13,4%) sem obstrução, 304 (38,0%) doença de um vaso, 169 (21,1%) doença de dois vasos e 220 (27,5%) doença de múltiplos vasos.

A maior parte dos fatores de risco cardiovascular (FRC) eram mais frequentes entre pacientes com doença de múltiplos vasos. Entretanto, frequências mais altas de fumantes atuais e pacientes com STEMI e níveis de LDL-C ligeiramente mais altos foram observados entre indivíduos com doença de um vaso, em comparação com aqueles com doença de múltiplos vasos. Também foi encontrada uma diferença significativa no histórico de DAC prévia entre os subgrupos: sem obstrução, 15 (15,6%); doença de um vaso, 57 (19,9%); doença de dois vasos, 36 (22,4%); e doença de múltiplos vasos, 74 (36,1%), com p<0,0001. Além disso, o histórico prévio de insuficiência cardíaca era mais frequente quanto mais alto era o nível de obstrução: sem obstrução, (24,5%); doença de um vaso, (13,6 %); doença de dois vasos, (13,5%); e doença de múltiplos vasos, (26,2%), com p=0,001. Da mesma forma, a fração de ejeção era mais baixa quanto mais alta era a gravidade da obstrução coronária, (mediana 59, FIQ: 43-66); doença de um vaso, (mediana 60, FIQ: 50-67); doença de dois vasos, (mediana 60, FIQ: 45-67); e doença de múltiplos vasos (mediana 51, FIQ: 41-65), p=0,001).

Em relação à terapia medicamentosa na admissão do hospital, pacientes com doença de um vaso tinham a menor porcentagem de administração de β bloqueadores (25,2%), comparados aos demais (p=0,048). Não foram observadas diferenças significativas em relação ao uso de medicamentos-padrão para DAC durante o monitoramento, independentemente do número de artérias coronárias principais obstruídas ( Tabela suplementar 1 ).

### Mortalidade e sobrevida

**Figura 1:**
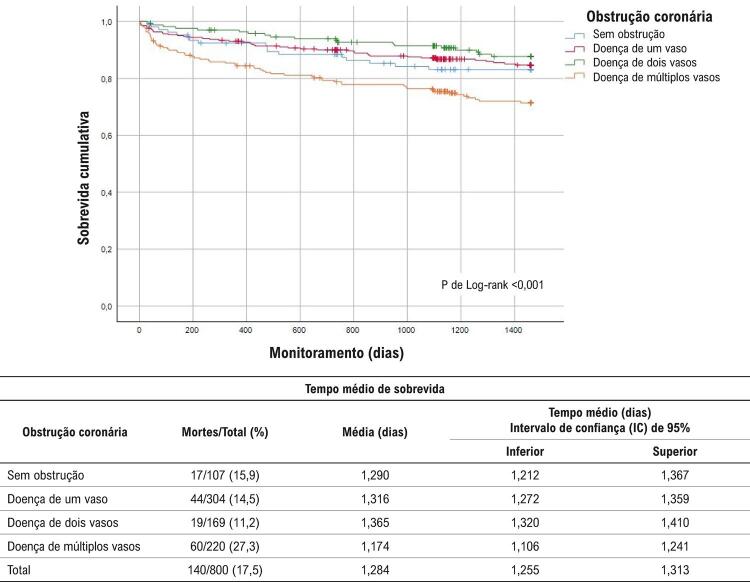
– *Curva de sobrevida de Kaplan Meyer para mortalidade global durante 4 anos de monitoramento*

**Figura 2:**
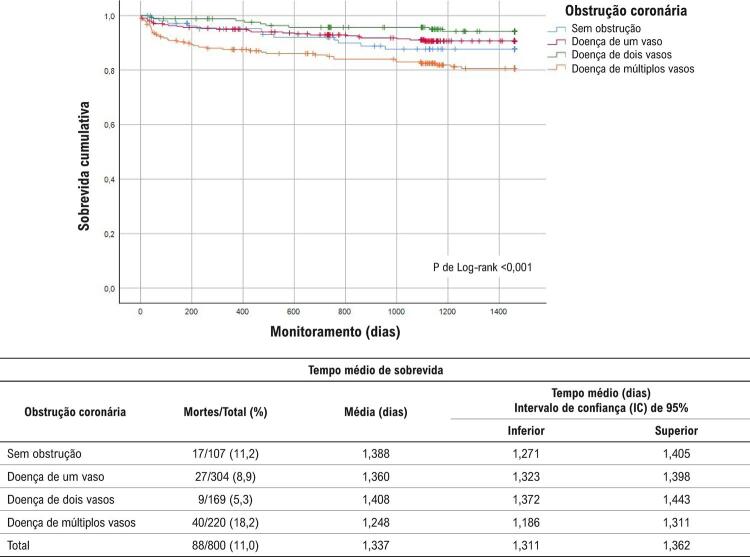
– *Curva de sobrevida de Kaplan Meyer para mortalidade por DCV durante 4 anos de monitoramento*

**Figura 3:**
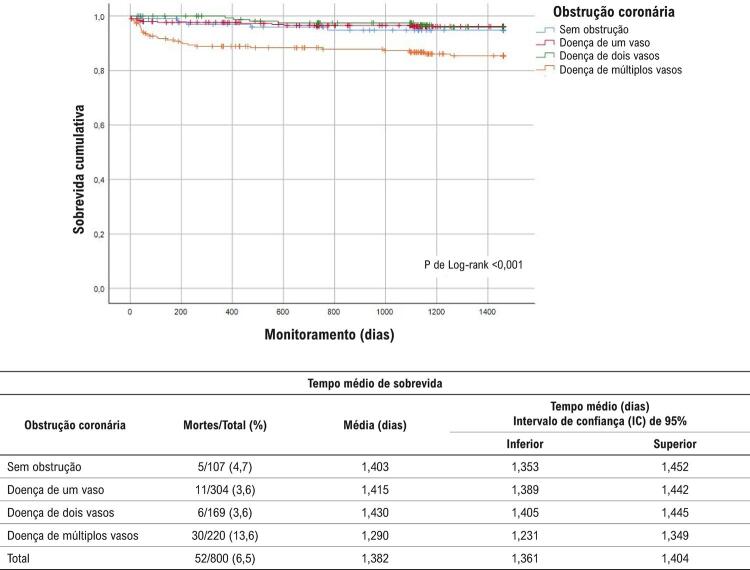
Curva de sobrevida de Kaplan Meyer para mortalidade por DAC durante 4 anos de monitoramento.

Em geral, foram observadas 140 mortes pós-SCA (88 mortes devido a DCV, das quais 52 eram devidas a DAC). Taxas de sobrevida mais baixas também foram detectadas entre indivíduos com a doença de múltiplos vasos (global, DCV e DAC, p de teste de Log-rank <0,0001) ( Figuras 1-3 ). Depois da padronização multivariada que incluiu idade, sexo e principais FRC, indivíduos com doença de múltiplos vasos ou com doença de um vaso tinham risco mais de duas vezes mais alto de mortalidade global em comparação aos sem obstrução, no monitoramento de 4 anos (Tabela 2).

Também foram encontradas RR mais altas (padronizadas por idade e sexo) para mortalidade por DCV aos 180 dias, e para mortalidade por DAC aos 180 dias, 1, 2 e 4 anos de monitoramento, entre os pacientes com doença de múltiplos vasos. Entretanto, após a padronização multivariada, não foram detectados riscos significativos de mortalidade por DCV ou DAC de acordo com a obstrução coronária durante o monitoramento (Tabelas 3 e 4). As análises de sensibilidade, exceto aquelas com STEMI, não alteraram a direção de nossos principais achados em relação à mortalidade global após 4 anos entre os pacientes com doença de um vaso [RR; 2,09 (IC 95%; 0,64-6,78); p = 0,22] e entre os com doença de múltiplos vasos [RR; 2,39 (IC 95%; 0,76-7,44); p = 0,13]. Outras padronizações quanto ao colesterol LDL, uso anterior de aspirina, medicamentos redutores de lipídeos, inibidores de enzima conversora da angiotensina (ECA), e β-bloqueadores não alteraram nossos achados principais.

## Discussão

No estudo ERICO, foi encontrado um risco mais alto de morte (mortalidade global) em ambos os subgrupos com doença de um vaso e doença de múltiplos vasos em comparação aos indivíduos sem obstrução (obstrução <50%) quatro anos após o evento agudo. Entre os pacientes com doença de múltiplos vasos, também foram observadas razões de risco mais altas para mortalidade por DCV e DAC, mas não após a padronização multivariável.

Os resultados estão de acordo com a maioria dos dados publicados sobre DAC que descreveram mortalidade alta e sobrevida baixa entre os pacientes com doença de múltiplos vasos.^7 - 9^ Entretanto, também foi descrita a alta mortalidade entre os portadores da doença de um vaso. Da mesma forma, Porter et al., descreveram o prognóstico de longo prazo entre a amostra de jovens adultos que foram submetidos a angiografia coronariana após um evento isquêmico.^22^ Este estudo descreveu um prognóstico comparável entre pacientes com doença de um vaso e os com doença de múltiplos vasos (pacientes com doença de um vaso tinham uma taxa de sobrevida mais baixa (63%) em relação à doença de múltiplos vasos (65%) p=0,001).^22^ Assim como em nossa amostra, a maior parte do participantes era do sexo masculino (88%) com frequência mais alta para fumantes atuais (58%). Talvez essas semelhanças possam ter contribuído para resultados semelhantes em ambos os coortes.

Ao analisar os fatores de risco da linha de base que possam ter levado a um prognóstico de longo prazo para pacientes com doença de um vaso, observaram-se as frequências mais altas de STEMI, fumantes atuais, e a frequência mais baixa de usuários de betabloqueadores na admissão hospitalar no estudo ERICO. Nosso estudo mostra semelhanças com outros estudos que apresentaram uma taxa de mortalidade mais alta associada ao tabagismo na presença de DAC. No estudo de Yudi et al., que foi realizado em indivíduos com SCA, aqueles que continuaram a fumar têm um risco 80% mais alto de sobrevida baixa, enquanto os que pararam de fumar têm sobrevida comparável às daqueles que nunca fumaram.^23^ Embora não se tenham informações sobre tabagismo durante o monitoramento, o status de tabagismo pode levar ao mau prognóstico entre os participantes com doença de um vaso.

Ao se analisar os medicamentos tomados, na linha de base, particularmente os pacientes com doença de um vaso tomaram menos β-bloqueadores do que aqueles com doença de múltiplos vasos (25,2% vs. 28,1%, p=0,048). Em um estudo brasileiro realizado por Nicolau et al., a administração precoce de β-bloqueadores na admissão hospitalar diminuiu a taxa de sobrevida sob monitoramento de longo prazo. Nesse estudo, demonstrou-se que a administração de β-bloqueadores nas primeiras 24 horas em pacientes NSTEMI contribuiu para um prognóstico melhor no longo prazo: tempo médio de sobrevida mais alto (11,86 ±0,4 anos vs. 9,92 ±0,39 anos, p<0,001).^24^ Além disso, outro estudo multicêntrico brasileiro mostra que a prevenção secundária às DAC de acordo com diretrizes está associada a renda mais alta e melhor acesso aos serviços de saúde. Em geral, a população brasileira que vive com renda baixa-média tem algumas barreiras para acessar os serviços de saúde públicos. Além disso, como mencionado anteriormente, os participantes do ERICO vieram de um bairro marcado por grande desigualdade.^25^

Além disso, indivíduos com doença de um ano, que tiveram a frequência mais baixa de usuários de β-bloqueadores e a frequência mais alta de fumantes na linha de base, tiveram o subtipo mais grave de SCA (STEMI). A análise de sensibilidade, com exceção dos pacientes com STEMI, resultou em um risco não significativo de mortalidade entre aqueles com doença de um vaso. Embora este estudo tenha considerado subtipo de SCA, tabagismo e uso de β-bloqueadores como variáveis de confusão nos modelos de regressão de Cox, não se pode excluir a possibilidade de efeito residual da baixa adesão e baixo controle de FRC poderia interferir no alto risco de mortalidade entre os indivíduos com doença de um vaso no estudo ERICO.

Além disso, o prognóstico de DAC também está relacionado à área do miocárdio em risco, e analisando a artéria coronária mais atingida em pacientes com doença de um vaso, identificou-se que 45,4% dos casos envolveram a artéria descendente anterior (DA). A DA é responsável por alimentar uma grande parte do miocárdio e, portanto, o fato de que pacientes com doença de um vaso têm uma alta porcentagem de obstrução dessa artéria coronária pode ter levado a um prognóstico pior nesses pacientes.

Como nossos resultados diferem daqueles encontrados em outros estudos, em sua maioria realizados no cuidado terciário em países desenvolvidos,^4 - 6^ em relação aos pacientes com doença de um vaso, a comparação de taxas de mortalidade de acordo com o número de artérias coronárias principais pós-SCA deve ser interpretada com prudência. Não há diferenças entre como as DAC obstrutivas podem ser classificadas. Além disso, também há diferenças na seleção de pacientes e nas opções de tratamento oferecidas nos hospitais. Além disso, o advento da tecnologia do tratamento nas últimas décadas pode ser parcialmente responsável pelos resultados diferentes obtidos.

Este estudo tem alguns pontos fortes. Ele oferece evidências consistentes sobre a relação entre o maior número de principais artérias coronárias com DAC, mortalidade mais alta e taxas de sobrevida mais baixas. Nosso estudo relatou informações sobre o prognóstico para pacientes com doença de um vaso que precisam ser entendidas como não sendo tão benignas quanto parecem. Esse fato reforça a importância de tratamento adequado e controle de fatores de risco cardiovascular após o evento SCA. A população do estudo ERICO tem nível socioeconômico baixo e é atendida em um hospital público, porém com a possibilidade de ser transferida para um centro de referência em cardiologia sem dificuldade. Além disso, monitoramos os medicamentos indicados para o tratamento da SCA em um período de um ano e avaliamos o consumo de acordo com a extensão da doença obstrutiva. Todos esses fatores, juntamente com o número significativo de pacientes em nosso estudo e o período de monitoramento de quatro anos, proporcionam uma oportunidade única para avaliar a associação entre taxas de mortalidade (global, DCV e DAC) de acordo com a gravidade da doença coronária quatro anos após o evento agudo. Entretanto, é necessário destacar algumas limitações. A angiografia invasiva para o diagnóstico de obstrução coronária não foi realizada por um profissional individualmente ou uma equipe restrita de profissionais, e isso pode ter gerado uma fonte de viés. Entretanto, um cardiologista do estudo ERICO revisou todos os casos e realizou a classificação de acordo com a extensão da doença obstrutiva.

## Conclusão

No estudo ERICO, a doença de múltiplos vasos, bem como a doença de um vaso, apresentaram uma mortalidade global alta em longo prazo após a SCA. Portanto, este estudo reforça a importância de se desenhar uma abordagem melhor para controlar e tratar pacientes em todas as faixas de risco cardiovascular, incluindo aqueles que eram aparentemente de baixo risco, atendidos no cuidado secundário.

## * Material suplementar

Para informação adicional, por favor, clique aqui:


