# Supplementary material for: O Prognóstico da Doença Arterial Coronariana em um Hospital Público no Brasil: Achado do Estudo ERICO
Source: Arq Bras Cardiol. 2021 Sep 16;117(5):978–85. [Article in Portuguese] doi: 10.36660/abc.20200399 (PMC8682093; doi:10.36660/abc.20200399)
Supplement: Supplementary file 1 [file 2020-0399_material_suplementar.pdf]

Supplemental table 1. Pharmacological treatment in the study sample (n=800) from the ERICO cohort according to coronary obstruction					
**Pharmacological treatment (%)**	**No obstruction**	**1 vessel-disease**	**2-vessel-disease**	**Multivessel-disease**	**P-value**
**Hospital admission for ACS**					
Aspirin	30 (29.7)	85 (29.7)	61 (37.9)	69 (33.0)	0.32
Clopidogrel	1 (1.0)	8 (2.8)	10 (6.2)	5 (2.5)	0.07
^{2} blocker	33 (32.7)	72 (25.2)	60 (37.3)	59 (28.1)	0.048
Calcium blocker	9 (9.0)	24 (8.4)	19 (11.8)	32 (15.2)	0.10
ACE inhibitor	39 (38.6)	118 (41.3)	69 (43.0)	80 (38.1)	0.78
ARB	6 (5.9)	15 (5.2)	11 (6.8)	11 (5.2)	0.90
Warfarin	1 (1.0)	4 (1.4)	1 (0.6)	7 (3.3)	0.20
Lipid lowering drugs	21 (20.8)	64 (22.4)	43 (26.7)	55 (26.2)	0.54
Fibrate	3 (3.0)	4 (1.4)	3 (1.9)	2 (1.0)	0.59
**30 days after ACS**					
Aspirin	74 (77.1)	221 (79.0)	126 (81.8)	146 (74.5)	0.41
Clopidogrel	42 (43.8)	137 (49.0)	77 (50.0)	84 (43.0)	0.436
^{2} blocker	58 (60.4)	170 (60.7)	103 (67.0)	127 (64.8)	0.538
Calcium blocker	8 (8.3)	42 (15.0)	23 (15.0)	26 (13.3)	0.395
ACE inhibitor	66 (68.8)	166 (59.3)	86 (55.8)	114 (58.2)	0.220
ARB	4 (4.2)	15 (5.4)	10 (6.5)	12 (6.1)	0.866
Warfarin	1 (1.0)	9 (3.2)	4 (2.6)	6 (3.1)	0.716
Statins	67 (69.8)	192 (68.6)	109 (70.8)	141 (72.0)	0.881
Fibrate	1 (1.0)	1 (0.4)	2 (1.3)	2 (1.0)	0.725
**180 days after ACS**					
Aspirin	72 (83.7)	213 (83.5)	130 (90.0)	141 (80.1)	0.192
Clopidogrel	16 (18.6)	74 (29.0)	43 (29.5)	48 (27.3)	0.262
^{2} blocker	53 (61.6)	156 (61.2)	109 (74.7)	122 (69.3)	0.028
Calcium blocker	8 (9.3)	46 (18.0)	25 (17.1)	28 (16.0)	0.288
ACE inhibitor	61 (71.0)	148 (58.0)	87 (59.6)	104 (59.1)	0.192
ARB	8 (9.3)	26 (10.2)	26 (17.8)	20 (11.5)	0.108
Warfarin	4 (4.7)	10 (4.0)	9 (6.2)	6 (3.4)	0.648
Statins	65 (75.6)	196 (77.0)	125 (85.6)	135 (76.7)	0.135
Fibrate	0 (0)	3 (1.2)	2 (1.4)	3 (1.7)	0.694
**1-year after ACS**					
Aspirin	63 (72.4)	203 (77.8)	113 (77.4)	136 (76.8)	0.776
Clopidogrel	12 (13.8)	48 (18.5)	29 (20.0)	32 (18.1)	0.703
^{2} blocker	51 (58.6)	155 (59.5)	96 (65.8)	113 (63.8)	0.513
Calcium blocker	8 (9.2)	37 (14.2)	23 (15.8)	30 (17.0)	0.389
ACE inhibitor	47 (54)	138 (53.0)	77 (52.7)	96 (54.2)	0.990
ARB	6 (7.0)	31 (12.0)	21 (14.5)	26 (14.7)	0.280
Warfarin	3 (3.4)	9 (3.4)	3 (2.1)	7 (4.0)	0.786
Statins	58 (66.7)	181 (69.3)	107 (73.3)	128 (72.3)	0.658
Fibrate	0 (0)	6 (2.3)	2 (1.5)	4 (2.3)	0.505

ACS: acute coronary syndrome
ACE: inhibitor: Angiotensin converting enzyme inhibitor
ARB: Angiotensin II converting enzyme inhibitor
