# Supplementary material for: O Prognóstico da Doença Arterial Coronariana em um Hospital Público no Brasil: Achado do Estudo ERICO
Source: Arq Bras Cardiol. 2021 Sep 16;117(5):978–85. [Article in Portuguese] doi: 10.36660/abc.20200399 (PMC8682093; doi:10.36660/abc.20200399)
Supplement: Translation

# Translation: The Prognosis of Coronary Artery Disease in a Brazilian Community Hospital: Findings from the ERICO Study

**Authors:** Tatiana Cristina Bruno, Marcio Sommer Bittencourt, Alessandra V. L. Quidim, Itamar Santos, Paulo Lotufo, Isabela Bensenor, Alessandra Goulart

**Affiliations:** 1 Brazil Universidade de São Paulo, Hospital Universitário de São Paulo - Centro de Pesquisa Clínica e Epidemiológica, São Paulo, SP - Brazil

**Keywords:** Survivorship, Mortality, Acute Coronary Syndrome, Coronary artery disease, Public Hospital, Epidemiology

## Abstract

**Background:**

Long-term prognosis post-acute coronary syndrome (ACS) in secondary care is not well-known. The severity of coronary artery disease (CAD) as a predictor of long-term mortality was evaluated in a community hospital in Brazil.

**Objective:**

We aimed to compare short and long-term prognosis after an ACS event according to severity of obstructive disease in patients attended in a secondary community hospital from prospective CAD cohort in Brazil (the Strategy of Registry of Acute Coronary Syndrome, ERICO study).

**Methods:**

Survival analyses were performed by Kaplan-Meier curves and Cox proportional hazard models (hazard ratios (HR) with respective 95% confidence interval (CI) to evaluate cumulative all-cause, CVD and CAD mortality according the coronary artery obstruction: no-obstruction (reference group), 1-vessel-disease, 2-vessel-disease, multivessel-disease) among 800 adults from an ERICO study during a 4-year-follow-up. HR are presented as crude and further adjusted for potential confounders from 180 days to 4-year follow-up after ACS. A p-value <0.05 was considered statistically significant.

**Results:**

Poorer survival rates were detected among individuals with multivessel-disease (all-cause, CVD and CAD, p-log rank< 0.0001). After multivariate adjustments, multivessel-disease |(HR; 2.33 (CI 95%; 1.10-4.95)) and 1-vessel-disease obstructed (HR; 2.44 (CI 95%; 1.11-5.34)) had the highest risk for all-cause mortality compared to those with no obstruction at 4-year follow-up.

**Conclusions:**

Not only multivessel-disease, but also 1-vessel-disease patients showed a high long-term mortality risk post-ACS. These findings highlight the importance of having a better approach in the treatment and control of cardiovascular risk even in apparently low-risk individuals attended to in secondary care.

## Introduction

Cardiovascular disease (CVD), particularly coronary artery disease (CAD), is still the main cause of death worldwide, including in Brazil.^1 , 2^Acute Coronary Syndrome (ACS), which includes unstable angina (UA), acute myocardial infarction (MI) with segment elevation (STEMI) and non-ST elevation myocardial infarction (NSTEMI), places a substantial burden on low- to middle-income countries, including Brazil.^3^National statistics reveal a higher burden of mortality among those with lower social strata, working, and younger age populations when compared to more affluent populations.^2 , 3^Most data reporting long-term prognosis in CAD comes from prospective studies performed in developed countries.^4 - 6^In those studies from specialized centers with tertiary care level cardiology units, higher long-term mortality rates were described among those with a higher number of obstructed arteries and CAD severity when compared to those patients with no obstruction (<50%).^7 - 9^In this scenario, long-term survival after an ACS event is not well-known among patients evaluated in secondary and primary care. Moreover, the lack of access to more specialized cardiologic approach and treatment after an acute coronary event is a huge public healthcare problem, particularly in developing countries. For instance, previous studies have already indicated a worse prognosis in CAD patients admitted into primary and secondary care who were not referred to specialized care.^10 - 12^The same is true for Brazil, where the difficulties to access tertiary care also seems to be responsible for higher mortality rates.^13^Thus, this study sought to compare short and long-term prognosis after an ACS event according to the severity of obstructive disease in patients attended to at a secondary community hospital from a prospective CAD cohort in Brazil (the Strategy of Registry of Acute Coronary Syndrome, ERICO study).

## Methods

### Sample design and population

All patients were participants in the ERICO study, a prospective cohort of ACS individuals recruited at the University Hospital from the University of São Paulo (HU-USP, in Portuguese) from February 2009 to December 2013. Further details about the ERICO study are presented elsewhere.^14^

In brief, the ERICO study is an ongoing cohort from HU-USP, a secondary community hospital with 260 hospital beds in the district of Butantã, which ha a population of 428,000 inhabitants in 2010.^15 , 16^Although Butantã has some socioeconomic indicators above the city’s average (e.g., average family income), it is a region characterized by broad inequalities.^16^

Here, the present study evaluated all participants (n=800/1085, 73.7%), admitted to the emergency department of HU-USP, with confirmed ACS submitted to invasive angiography for the diagnosis of coronary obstruction and posterior clinical decision after acute phase (exclusive clinical treatment, percutaneous coronary intervention (PCI) or coronary artery bypass grafting (CABG)). All exams were performed during the acute phase of a coronary event in our main cardiologic referral center, *Instituto do Coração* (InCor), a reference center in cardiology nearly eight kilometers from HU-USP. Since HU-USP is a non-specialized hospital, there is no availability of cardiac catheterization procedures or CABG.

### Definition of Acute Coronary Syndrome (ACS)

All patients with suspected ACS at the emergency department of HU-USP were screened to participate in the ERICO study. The eligibility for taking part in the ERICO study requires the patient to be diagnosed as having an ST elevation myocardial infarction (STEMI), a non-ST elevation myocardial infarction (NSTEMI), or unstable angina (UA); the criteria used to define ACS were:^14 - 17^

1. Myocardial infarction: the presence of symptoms consistent with cardiac ischemia within 24 hours of hospital admission and troponin I levels above the 99^th^percentile with a test-specific coefficient of variation < 10%.
  1a) ST elevation myocardial infarction: the presence of criteria for coronary artery disease (CAD) plus one of the following: persistent ST segment elevation equal to or greater than 1mm in two contiguous electrocardiographic leads or the presence of a new or presumably new left bundle branch block.
  1b) Non-ST elevation myocardial infarction: the presence of criteria for myocardial infarction but not STEMI.
2. Unstable angina: symptoms consistent with cardiac ischemia 24 hours prior to hospital admission, absence of myocardial infarction criteria, and at least one of the following: history of coronary artery disease; positive coronary disease stratification test (invasive or noninvasive); transient ST segment changes equal to or greater than 0.5mm in two contiguous leads, new T-wave inversion equal to or greater than 1mm and/or pseudonormalization of previously inverted T-waves; troponin I equal to or greater than 0.4 ng/ml (which guarantees troponin I levels above the 99^th^percentile regardless of the utilized kit); or diagnostic concordance of two independent doctors.

### Coronary artery disease classification

The classification of coronary disease was based on the presence of ≥50% obstruction of at least 1 major coronary artery or any of its major branches: anterior descending artery (AD), circumflex artery (CX), and right coronary artery (RCA). The following categories of coronary obstruction was made up of: Group 1: no obstruction when all vessels had <50% obstruction, Group 2: 1-vessel-disease when ≥50% obstruction was present in one major coronary artery or any of its major branches, Group 3: 2-vessel-disease coronary obstruction ≥50% in two major coronary arteries or its major branches, and Group 4: multivessel-disease with obstruction in all three major coronary artieries (or its major branches) or Left Main(LM) ≥50% obstruction or presence of previous coronary artery bypass grafting (CABG).

### Study Protocol

Upon hospital admission for ACS, after having signed the informed consent form, all participants provided baseline information based on standardized questionnaires that included sociodemographic data, main cardiovascular risk factors (hypertension, diabetes, obesity, dyslipidemia, smoking, personal or family history of coronary artery disease, physical inactivity, cocaine use, and menopause) and the use of previous medication. Clinical conditions were self-reported.

Three physicians were independently responsible for reviewing information and validating ACS cases. The study protocol also included a blood sample for laboratory testing, such as: troponin I, MB-creatine kinase, hemogram, and lipid profile (including total cholesterol, HDL and LDL- cholesterol (C), and triglycerides).

After 30 days of the acute event, all participants were invited to update their information about cardiovascular risks. At six months and annually after the initial event, patients were contacted by phone to update their information, their vital status, cardiovascular history, and medication use. Whenever a patient reported a new potential ACS event, an investigation was initiated to acquire further information. ERICO has been described in detail elsewhere.^14^

## Results

Information on the three fatal endpoints: all-cause, CVD and CAD mortality were record by the ERICO study. Vital status was updated through medical records and death certificates. Mortality data was confirmed by official death certificates in collaboration with the city of São Paulo’s health statistics system (PRO-AIM, Program for Improvement of Mortality Information in the Municipality of São Paulo) and State’s health offices (SEADE foundation, Healthcare Data Analysis System of the State of São Paulo’s Health) and the Brazilian Ministry of Health. On a regular basis, the research team prepared a list of individuals who were reported as dead or with whom contact had been lost. State and municipal health agencies searched their databases for death certificates reporting results to the ERICO study research team. In the present study, the basic cause of death was used. Two physicians independently analyzed the death certificate and, when necessary, the underlying cause of death was reclassified. If there was disagreement between them, a third doctor performed the analysis of the death certificate, followed by a discussion and consensual decision. Participants were defined to have died from cardiovascular cause (“cardiovascular mortality”) when the cause of death could be classified as “Diseased of Circulatory System”, according to the International Classification of Diseases, version 10 (ICD-10), chapter IX, or if the cause of death was identified following ICD-10 code R57.0 “Cardiogenic Shock”. Each identified event was adjudicated using predefined international criteria.^18 , 19^Participants’ mortality was classified as “post-IM mortality” whenever fatal CAD was identified as the main cause of death. For CAD as the cause of death, the definition of myocardial infarction (I21.X) was used, which was also present in Chapter IX of circulatory diseases of the ICD-10. All-cause mortality refers to the deceases regardless of underlying causes.

The study protocol was approved by the Institutional Review Board addressing research in human beings. All subjects provided a written informed consent form for the study.

### Statistical Analysis

Descriptive analyses of ERICO participants were presented according to the predefined groups of coronary obstruction described above. Categorical variables, presented in absolute and relative frequencies, were analyzed using the chi-squared test. As no parametric distribution was observed by a normality test of Kolmogorov-Smirnov, continuous variables are presented as median values with a respective interquartile range (IQR) and the distribution among coronary obstruction subgroups were compared using Kruskal-Wallis tests.

Survival analyses were performed by applying Kaplan-Meier curves^20^and Cox proportional hazards models (hazard ratios (HR) with respective 95% CI)^21^to evaluate cumulative all-cause, CVD, and CAD mortality according the number of obstructed major coronary arteries or any of their major branches (no-obstruction: reference group, 1-vessel-disease, 2-vessel-disease, multivessel-disease). For all patients in this sample there was a 7-year follow-up period, with the median follow up time of 1,460 days, corresponding to 4 years. Therefore, we opted to do Cox Regression analysis and Hazard Ratio in 180 days and yearly up to 4 years after an acute event. The Cox regression models were calculated as follows: crude, adjusted for age-sex, and the full model adjusted for the history of the previous CAD, ACS subtype (UA, NSTEMI, STEMI), smoking (past, current, and never), hypertension, diabetes, dyslipidemia, and type of procedure performed (medical therapy, percutaneous or surgical). Additional models adjusted for LDL-cholesterol, previous use of aspirin, lipid-lowering drugs, angiotensin-converting enzyme inhibitor (ACE), and β-blocker were also evaluated.

All tests were two-tailed with a significance of <0.05. All statistical analyses were performed using the statistics program, SPSS® Statistics, version 25.0, made available by IBM®.

## Results

### Casuistic

Of the 800 participants who underwent invasive angiography (February 2009 and December 2013), 343 (42.9%) underwent conservative treatment, including at least three of the following medications: aspirin, β blocker, ACE inhibitor or angiotensin II converting enzyme inhibitor, and lipid lowering medications (statin or fibrate). Among those under conservative treatment, 15 (4.4%) underwent chemical thrombolysis. Regarding invasive therapeutic strategies, 400 participants (50.0%) underwent percutaneous coronary intervention (PCI) with a stent implant (75.8% metal stent, 13.3% balloon angioplasty, 10.9% drug-eluting stent) and 57 (7.1%) underwent CABG.

### Clinical and sociodemographic characteristics

Clinical and sociodemographic characteristics according to the number of obstructed major coronary arteries are shown in Table 1. The presence of obstructed major coronary arteries was as follows: 107 (13.4%) with no obstruction, 304 (38.0%) 1-vessel-disease, 169 (21.1%) 2-vessel-disease, and 220 (27.5%) multivessel-disease.

Most cardiovascular risk (CVRF) were more frequent among those patients with multivessel-disease. However, higher frequencies of current smokers, STEMI and slightly higher levels of LDL-C were noticed among individuals with 1-vessel-disease when compared to those with multivessel-disease. A significant difference was also found in the history of previous CAD across subgroups: with no obstruction, 15 (15.6%); 1-vessel-disease, 57 (19.9%); 2-vessel-disease, 36 (22.4%); and multivessel-disease, 74 (36.1%), with p<0.0001. Further, the higher the level of obstruction, the more frequent the previous history of heart failure: no obstruction (24.5%), 1-vessel-disease (13.6 %), 2-vessel-disease (13.5%). and multivessel-disease (26.2%), with p=0.001. Likewise, the higher the severity of coronary obstruction, the lower the ejection fraction: with no obstruction (median 59, IQR: 43-66), 1-vessel-disease (median 60, IQR: 50-67), 2-vessel-disease (median 60, IQR:45-67), and multivessel-disease (median 51, IQR: 41-65), p=0.001).

Regarding drug therapy upon hospital admission, patients with 1-vessel-disease had the lowest percentage of β blocker administration (25.2%) when compared to the others (p=0.048). No significant differences were identified regarding standard medication use for CAD during follow-up, regardless of the number of obstructed major coronary arteries ( Supplemental Table 1 ).

### Mortality and survival

Overall, the present study observed 140 deaths post-ACS (88 deaths due to CVD, of which 52 were due to CAD). The poorer survival rates were also detected among individuals with multivessel-disease (all-cause, CVD, and CAD, p-log rank< 0.0001) ( Figures 1-3 ). After multivariate adjustment that included age, sex, and main CVRF, either individuals with multivessel disease or those with 1-vessel-disease had a higher risk of more than twice for all-cause mortality as compared to those without obstruction at 4-year follow-up (Table 2).

**Figure 1:** – *Kaplan Meyer survival curve for all-cause mortality during 4 years of follow-up.*

**Figure 2:** – *Kaplan Meyer survival curve for CVD mortality during 4 years of follow-up.*

**Figure 3:** Kaplan Meyer survival curve for CAD mortality during 4 years of follow-up.

We also found higher HRs (adjusted by age and sex) for CVD mortality at 180 days and for CAD mortality at 180 days, and 1, 2, and 4 years of follow-up among those with multivessel disease. However, after multivariate adjustments, no significant risks were detected for CVD and CAD mortality according coronary obstruction during the follow-up (Tables 3 and 4). Sensitivity analysis, excluding those with STEMI, did not change the direction of our main findings regarding all-cause mortality after 4 years among those with 1-vessel-disease [HR; 2.09 (CI 95%; 0.64-6.78); p = 0.22] and for those with multivessel-disease [HR; 2.39 (CI 95%; 0.76-7.44); p = 0.13]. Further adjustments for LDL-cholesterol, previous use of aspirin, lipid-lowering drugs, angiotensin-converting enzyme inhibitor (ACE), and β-blocker did not change our main findings.

## Discussion

In the ERICO study, our study found a higher risk of death (all-cause mortality) in both subgroups with 1-vessel-disease and multivessel-disease compared to individuals with no obstruction (< 50% obstruction) four years after the acute event. Among those with multivessel-disease, higher hazard ratios for CVD and CAD mortality were also observed but not after the multivariable adjustment.

Our results are in accordance with most data published in CAD that described high mortality and poor survival among patients with multivessel disease.^7 - 9^However, our study also described high mortality among those with 1-vessel disease. Similarly, Porter et al. described the long-term prognosis within a sample of young adults who underwent a coronary angiography after an ischemic event.^22^This study described comparable prognosis among patients with a 1-vessel-disease with those with multivessel-disease (1-vessel-disease had a lower survival rate (63%) vs. multivessel-disease (65%) p=0.001).^22^As in our sample, most participants were male (88%), with a higher frequency of current smokers (58%). These similarities may well have contributed to similar results in both cohorts.

By reviewing baseline risk factors that may have led to worse long-term prognosis for 1-vessel-disease patients, this study observed the highest frequencies of STEMI and current smokers, and the lowest frequency of beta-blocker users upon hospital admission in the ERICO study. Our study shows similarity with other studies that showed a higher mortality rate when associated with smoking in the presence of CAD. In the study of Yudi et al., which was performed with individuals with ACS, those who continued to smoke have an 80% risk of lower survival, while those who quit showed a survival rate comparable to lifelong non-smokers.^23^Although information about smoking during follow-up is scarce, the smoking status can lead to a poor prognosis among 1-vessel-disease participants.

When the medication was analyzed, at baseline, 1-vessel-disease patients in particular have taken less β-blockers than those with multivessel-disease (25.2% versus 28.1%, p=0.048). In a Brazilian study conducted by Nicolau et al., the early administration of β-blockers during hospital admission decreased the survival rate in a long-term follow-up. This study showed that β-blocker administration within the first 24 hours in NSTEMI patients contributed to a better prognosis over the long-term: higher mean survival time (11.86 ±0.4 years vs 9.92 ±0.39 years p<0.001).^24^Furthermore, another Brazilian multicenter study showed that the secondary prevention to CAD according to guidelines is linked to higher income and better access to health services. Overall, most of the Brazilian population living with a lower-middle income has some barriers to access public health care services. Moreover, as previously mentioned, the ERICO participants come from a neighborhood characterized by broad inequalities.^25^

In addition, 1-vessel-disease individuals, who were the lowest frequency of β-blocker users and the highest frequency of smokers at baseline, had the most severe subtype of ACS (STEMI). Sensitivity analysis, excluding those with STEMI, resulted in a non-significant mortality risk among those with 1-vessel. Although our study considered the ACS subtype, smoking, and β-blocker use as confounding variables in the Cox regression models, one cannot rule out the possibility that a residual effect of low adherence and poor control of CVRF could interfere in the high risk of mortality among individuals with only 1-vessel-disease in the ERICO study.

Moreover, the prognosis of CAD is also related to the area of the myocardium at risk and analyzing the most affected coronary artery in patients with 1-vessel-disease, this study found that 45.4% of the cases involved the anterior descending artery (AD). The AD is responsible for supplying a large part of the myocardium; therefore, the fact that 1-vessel-disease patients have a high percentage of obstruction of this coronary artery may have led to a worse prognosis in those patients.

Since our results differ from those found in other studies, mostly performed in tertiary care in developed countries,^4 - 6^regarding patients with 1-vessel-disease, the comparison of mortality rates according to the number of major coronary arteries in post-ACS must be interpreted with prudence. There are differences in how obstructive CAD can be classified. Furthermore, there are also differences in the selection of patients and treatment options offered at hospitals. Moreover, the advent of technology in treatment in recent decades may be partially responsible for the differing results in our study.

Our study has some strengths. It provides consistent evidence about the relationship between the larger number of major coronary arteries with CAD, higher mortality, and lower survival rates. Our study reported information of prognosis for 1-vessel-disease that needs to be considered less benign than they seem. This fact reinforces the importance of adequate treatment and control of cardiovascular risk factors after an ACS event. The ERICO population study has low socioeconomic level and was attended to at a community hospital, but with the ability to transfer patients to a specialized cardiology referral center without difficulty. In addition, we monitored the medications indicated for the treatment of ACS over a period of one year and evaluated the intake according to the extent of the obstructive disease. All of these factors, coupled with the significant number of patients in our study and the four-year follow-up time frame provides a single opportunity to evaluate the association among mortality rates (all-cause, CVD, and CAD) according to the severity of coronary disease four years after the acute event. Nonetheless, some limitations need to be pointed out here. Invasive angiography for the diagnosis of coronary obstruction was not performed by a single or a restricted team of professionals which might have generated a source of bias. However, a cardiologist from the ERICO study revised all cases and performed the classification according the extension of the obstructive disease.

## Conclusion

In the ERICO study, multivessel-disease, as well as 1-vessel-disease, presented high long-term all-cause mortality after ACS. Therefore, our study reinforces the importance of designing a better approach to controlling and treating patients within all cardiovascular risk ranges, including those at apparently low risk attended to in secondary care.

## * Supplemental Materials

For additional information, please click here:
